# Ethical Dilemmas and Approaches to Acute Pain Management in Patients With Substance Use Disorders

**DOI:** 10.7759/cureus.95044

**Published:** 2025-10-21

**Authors:** Trevor Virno, Carl Van Rensburg, Marcia Ballantyne

**Affiliations:** 1 College of Medicine, Lake Erie College of Osteopathic Medicine, Bradenton, USA; 2 Pathology, Lake Erie College of Osteopathic Medicine, Bradenton, USA

**Keywords:** acute pain management, beneficence, medical ethics, multimodal analgesia, multi-modality pain management, opioid addiction, opioid free pain management, opioid pandemic, perioperative care, relapse prevention

## Abstract

The opioid epidemic has intensified ethical dilemmas in acute pain management, particularly for patients with opioid use disorder (OUD). Clinicians face the dual responsibility of relieving pain while minimizing the risks of relapse and misuse. Historically, this population has been vulnerable to undertreatment due to stigma and fear of addiction, yet inadequate analgesia itself can worsen outcomes and undermine recovery. This review integrates ethical principles with clinical evidence to propose a framework for managing acute pain in patients with OUD.

Ethical principles of autonomy, beneficence, nonmaleficence, and justice must guide care while balancing effective analgesia and addiction stability. Regional anesthesia and multimodal approaches are evaluated as ethically compelling alternatives to opioid-centered regimens, offering effective pain relief while reducing potential harms. System-level barriers, including stigma, inconsistent protocols, and limited access to addiction expertise, hinder equitable care. Addressing these requires institutional and policy reforms that prioritize education, interdisciplinary collaboration, and expanded access to MOUD and regional anesthesia.

This review concludes that ethically sound acute pain management in OUD demands individualized, multimodal approaches that respect patient autonomy, prevent harm, and advance justice. Clinicians must integrate ethics and evidence to deliver compassionate, equitable care in the midst of the opioid crisis.

## Introduction and background

Managing acute pain in patients with opioid use disorder (OUD) presents a complex clinical and ethical challenge. As the opioid epidemic continues to claim tens of thousands of lives annually, with over 81,000 opioid-involved deaths reported in 2022 alone, clinicians are increasingly tasked with balancing adequate pain relief against the risk of relapse, overdose, and prolonged opioid use [1]. Patients with OUD frequently present for surgery or acute injury management and are at heightened risk for both undertreatment of pain and iatrogenic harm due to stigma, fragmented care, and outdated assumptions about opioid tolerance and addiction. This article examines the ethical and clinical motives involved in acute pain management for this vulnerable population. It argues that a multimodal, individualized, and stigma-free approach is not only evidence-based but also ethically necessary. Recent literature strongly supports the continuation of medications for opioid use disorder, such as buprenorphine and methadone, during the perioperative period [2]. Regional anesthesia, non-opioid adjuncts, and non-pharmacologic modalities such as transcutaneous electrical stimulation and aromatherapy have emerged as effective opioid-sparing strategies [3,4]. Despite the growing body of research, significant implementation gaps remain. Meta-analyses reveal persistent over-reliance on opioids postoperatively, while reviews emphasize the need for systems-level reform and additional research [5]. This review synthesizes the current evidence and proposes an ethical framework for acute pain care in patients with OUD, emphasizing respect for patient autonomy, minimization of harm, and equitable access to effective therapies.

## Review

Ethical and clinical controversies

The ethical management of acute pain in patients with OUD demands careful attention to the ethical principles of autonomy, beneficence, nonmaleficence, and justice. These patients often face dual stigmas: first, for their history of substance use, and second, when they seek care for pain, leading to undertreatment, distrust, and fragmented decision-making. A commitment to ethical care must begin with a recognition of these disparities and a resolve to address them through evidence-based, compassionate practice. Identifying which patients are most vulnerable to persistent opioid use after acute pain episodes is critical to preventing misuse, relapse, and long-term dependence, especially in those with OUD or other substance use vulnerabilities. A refined understanding of risk factors allows clinicians to target interventions more effectively and personalize pain management strategies.

Studies highlight specific patient characteristics associated with increased risk of prolonged opioid use after surgery or injury [6-8]. In a large retrospective cohort study of 6,432 patients undergoing total joint arthroplasty, current tobacco use, antidepressant therapy, post-traumatic stress disorder (PTSD), obesity, and co-occurring substance use disorders (SUDs) were all strongly linked to a higher risk of persistent opioid use [6]. These findings emphasize the role of psychological and behavioral health in pain trajectories, particularly in populations already predisposed to opioid misuse. Psychiatric comorbidities, including anxiety, depression, and trauma-related disorders, consistently appear as predictors of chronic opioid use. Hah et al. (2017) emphasized the need to integrate psychosocial and behavioral interventions early in the perioperative period to reduce long-term dependence [9]. Likewise, Hsu et al. (2019) advocated for non-pharmacologic approaches like cognitive-behavioral therapy, mindfulness, and even aromatherapy, recognizing that pain is not solely a physical experience but a biopsychosocial one [4].

Patients with chronic pain syndromes or those undergoing high-pain surgeries are also particularly vulnerable. Glare et al. (2019) noted that chronic post-surgical pain, especially when unresponsive to opioids, poses a dilemma that requires transition to non-opioid analgesics and consideration of alternative modalities [10]. Similarly, Dada et al. (2019) highlighted that rebound pain after peripheral nerve blocks can paradoxically increase opioid consumption postoperatively if not adequately anticipated and managed [7]. Importantly, structural and provider-level factors also influence vulnerability. Carley et al. (2021), in a meta-analysis of over 100 studies, concluded that despite mounting evidence for multimodal analgesia, little progress has been made in implementing strategies that reduce persistent opioid use [5]. This suggests that systemic inertia, provider bias, and gaps in education may allow unnecessary opioid exposure even in known high-risk groups.

Beyond clinical characteristics, the social determinants of health, housing instability, access to care, and systemic racism are often underrecognized contributors to opioid misuse vulnerability. While not always directly measured in clinical studies, these factors shape how patients experience pain, access alternatives, and adhere to treatment plans. Vulnerability to persistent opioid use is multifactorial, encompassing biological, psychological, and structural domains. Recognizing these risk factors allows for anticipatory planning, such as early initiation of multimodal analgesia, continuity of medications for opioid use disorder (MOUD), and targeted behavioral health interventions. Proactive strategies are not just clinically effective; they represent an ethical obligation to those most at risk of being failed by the current system.

One of the most complex and debated aspects of managing acute pain in patients with OUD is the perioperative management of MOUD, particularly buprenorphine and methadone. Historically, many clinicians have elected to pause MOUD to avoid concerns about analgesic ceiling effects or interference with full opioid agonists. However, emerging evidence and expert consensus are shifting toward continuation of MOUD, grounded in both clinical outcomes and ethical principles. Buprenorphine, a partial opioid agonist with high receptor affinity and ceiling respiratory depression effects, presents a unique challenge. Traditional practice often involved discontinuing buprenorphine preoperatively to allow full agonists to work more effectively. Yet this approach is increasingly questioned. Goel et al. (2019) strongly supported the continuation of buprenorphine through the perioperative period, especially when combined with regional anesthesia and non-opioid adjuncts [2]. Experts emphasized that interruption can lead to destabilization, increased risk of relapse, and complex transitions back onto MOUD.

Fiellin et al. (2022) offered a nuanced compromise: reducing buprenorphine/naloxone to 8-12 mg daily 24-36 hours before anticipated high-intensity pain [11]. This strategy preserves partial agonist receptor activity while allowing some analgesic benefit from additional short-acting opioids, minimizing withdrawal or destabilization. This middle-ground approach has gained traction in high-acuity settings such as orthopedic or oncologic surgeries. Methadone, a full opioid agonist with a long half-life, poses different considerations. Continuation during hospitalization is typically favored, particularly due to the risks associated with withdrawal and disruption of opioid tolerance. Importantly, missed doses of methadone can increase overdose risk due to loss of tolerance upon re-initiation.

The ethical implications of MOUD interruption are significant. Discontinuing life-saving treatment for the sake of short-term analgesic flexibility can violate the principles of nonmaleficence and justice. Patients with OUD are at high risk of overdose, destabilization, and stigma when MOUD is withheld. Furthermore, such disruptions can damage trust in healthcare providers and reduce future care-seeking behavior. Graff et al. (2018) reinforce that the goal is not to avoid opioids altogether, but to strategically layer multiple modalities, regional anesthesia, non-opioid medications like ketamine, and continued MOUD, to achieve analgesia without compromising addiction care [12]. Emerging evidence underscores the growing feasibility of this multimodal synergy, supported by reports from Schmedt et al. (2023) and Kumar et al. (2017) [3,13].

Finally, decisions around MOUD management should be individualized and guided by interdisciplinary collaboration. Engagement with addiction specialists, anesthesiologists, and surgeons ensures that pain and addiction care is a multimodal plan. Such integrated planning reinforces both the beneficence and autonomy of the patient. In summary, the paradigm is shifting continuation of MOUD, particularly buprenorphine and methadone, is increasingly viewed as safe, effective, and ethically superior when paired with multimodal pain strategies. Interrupting MOUD, once common, is now recognized as a high-risk decision that should be reserved for only the most exceptional cases, with careful justification and a plan for rapid reinstatement.

Synthesis and recommendations

Regional anesthesia and non-opioid adjuncts are critical components of multimodal analgesia and have emerged as indispensable tools in managing acute pain, particularly in patients with OUD. For these individuals, effective pain relief must be balanced with the minimization of systemic opioid exposure and the maintenance of MOUD, making opioid-sparing strategies even more essential. Several high-quality reviews and meta-analyses support the efficacy of regional anesthesia in reducing both acute pain scores and longer-term opioid use [3,7,8,13]. Schmedt et al. (2023), examining adults undergoing non-cardiac surgeries, demonstrated that regional techniques (e.g., peripheral nerve blocks, neuraxial anesthesia) reduced the risk of persistent opioid use by nearly 50%. Likewise, Pepper et al. (2024), in a systematic review of 37 studies, found that regional anesthesia significantly decreased the incidence of chronic postsurgical pain at up to six months postoperatively [8]. These findings suggest that regional blocks not only manage immediate pain but may also reduce the risk of chronic pain, a key driver of ongoing opioid use.

Regional techniques are especially valuable when combined with opioid-free or opioid-sparing anesthesia. De Cassai et al. (2022) explored this approach in pain-sensitive and oncologic surgeries, concluding that opioid-free anesthesia (OFA) is both feasible and effective when regional and nerve blocks are integrated into the anesthetic plan [14]. OFA is particularly attractive in OUD patients, as it can provide profound analgesia without triggering cravings or relapse.

However, regional anesthesia is not without limitations. Dada et al. (2019) noted the phenomenon of rebound pain, a sharp increase in pain as the block wears off, which can paradoxically increase opioid consumption if not properly managed [7]. To mitigate this, perioperative protocols should include scheduled non-opioid medications (e.g., NSAIDs, acetaminophen) timed to overlap with block resolution. Shams et al. (2022) also emphasized the importance of recognizing contraindications to regional anesthesia, such as infection at the injection site or coagulopathy, which may limit its applicability in select patients [15]. Beyond regional techniques, a wide array of non-opioid pharmacologic adjuncts has demonstrated efficacy in managing perioperative pain. Graff et al. (2018) highlighted agents such as ketamine, dexmedetomidine, IV acetaminophen, and ketorolac as effective opioid-sparing medications [12]. These agents act synergistically on different pain pathways and can be combined safely with MOUD, unlike traditional opioids, which may require more careful coordination. Hsu et al. (2019) and Kumar et al. (2017) both supported incorporating regional anesthesia and adjuncts into a multimodal strategy, stressing the importance of preoperative planning to ensure adequate coverage during peak pain periods and smooth transitions postoperatively [4,13].

In conclusion, regional anesthesia and non-opioid adjuncts are not simply alternatives to opioids; they are cornerstones of ethical and effective pain management in patients with OUD. When implemented thoughtfully, these strategies enhance analgesia, reduce opioid reliance, and support the overall stability of recovery-focused care.

OFA, patient autonomy, and clinician discomfort

The integration of OFA and reduced-opioid strategies in patients with OUD invites a range of ethical and clinical controversies. While the evidence base supporting multimodal, non-opioid approaches continues to grow, implementation is not always straightforward. Ethical dilemmas often emerge around patient autonomy, clinician risk aversion, and variability in evidence interpretation, factors that may inadvertently compromise care quality and equity.

A central tension lies in the growing momentum for opioid-free anesthesia, which seeks to eliminate intraoperative opioids entirely through the use of regional anesthesia, ketamine, magnesium, and lidocaine. De Cassai et al. (2022) affirmed the feasibility of OFA in pain-sensitive and oncologic surgeries, noting equivalent or superior pain control [14]. However, not all patients or surgeries are suitable candidates, and concerns around rebound pain or block failure remain. From an ethical perspective, promoting OFA aligns with nonmaleficence, avoiding harm through unnecessary opioid exposure, and beneficence, especially when regional anesthesia improves pain control and reduces long-term opioid use. Yet patient autonomy must be preserved. Patients should be fully informed of their options, including potential benefits and risks of OFA, and involved in decision-making. When patients request traditional opioid-based analgesia due to prior experiences or expectations, the ethical principle of respect for autonomy becomes paramount, even when this conflicts with the clinician's preference for opioid minimization.

Clinicians may also experience discomfort with OFA protocols, particularly in complex patients or unfamiliar procedural contexts. As Carvalho et al. (2019) notes, patients in pain may be especially vulnerable to persuasion or dismissal, heightening the moral obligation to communicate clearly and empathetically [16]. Clinician biases toward perceived "drug-seeking" behavior can lead to undertreatment of pain, particularly in individuals with a history of substance use. This is compounded by stigma, which subtly influences care decisions and erodes trust. Dowell et al. (2022) remind us of the stakes: in 2022 alone, over 81,000 opioid-related deaths occurred in the U.S., often among individuals marginalized in their care experiences [1]. Ultimately, clinicians must navigate this landscape with humility and patient-centeredness. As emphasized in the Graff et al. (2018) and Kumar et al. (2017) reviews, best practices hinge not only on pharmacologic strategies but also on collaborative, ethical care [12,13]. This includes involving patients in their own pain plans, accounting for psychological and social dimensions of pain, and recognizing that "opioid-free" is not synonymous with "better" in all cases.

In balancing innovation with compassion, the true ethical imperative is clear: manage pain effectively, preserve dignity, and ensure that OUD patients receive care free of bias, abandonment, or a lesser standard of care due to an undeserving stigma. The management of acute pain in patients with OUD requires an evidence-based, ethically grounded, and patient-centered approach. The reviewed literature provides a clear roadmap for achieving effective analgesia while minimizing harm, supporting recovery, and honoring patient autonomy. However, the pathway to implementation is complex and must navigate clinical variability, ethical tensions, and systemic challenges.

**Table 1:** Alternative and adjunctive options for acute pain management in patients with opioid use disorder

Category	Therapy	Mechanism/Approach	Ethical Consideration
Non-Opioid Pharmacologic Options	Acetaminophen	Central COX inhibition	First-line analgesic, minimal misuse potential
	NSAIDs (Ketorolac, Ibuprofen)	COX inhibition reducing inflammation	Reduces opioid requirements, caution with renal/GI risk
	Ketamine (low-dose infusion)	NMDA receptor antagonist	Effective in opioid-tolerant patients; supports beneficence
	Dexmedetomidine	Alpha-2 agonist, sedative-analgesic	Facilitates non-opioid analgesia with minimal respiratory depression
	Gabapentinoids (Gabapentin, Pregabalin)	Modulates calcium channels	May reduce hyperalgesia; consider sedation risks
Regional and Local Techniques	Peripheral Nerve Blocks	Local anesthetic blockade of targeted nerves	Proven opioid-sparing; prolongation via adjuvants
	Epidural/Spinal Anesthesia	Neuraxial blockade for lower body surgeries	High efficacy for surgical pain; requires expertise
	Local Wound Infiltration	Direct anesthetic application	Low-risk adjunct for minor surgeries
Adjunctive Non-Pharmacologic Strategies	Transcutaneous Electrical Nerve Stimulation (TENS)	Neuromodulation via skin electrodes	Reduces pain perception without pharmacologic risk
	Ice/Elevation	Physical reduction of inflammation	Non-invasive adjunct, enhances patient autonomy
	Aromatherapy/Relaxation	Anxiety reduction	Supports psychological comfort and holistic care
Medication for OUD (MOUD) Considerations	Continue Buprenorphine	Partial opioid agonist	Prevents relapse; maintains receptor stability
	Continue Methadone	Full agonist with long half-life	Avoids withdrawal, supports continuity of care
	Coordinate with Addiction Medicine	Integrated management	Upholds justice and beneficence through multidisciplinary care

Clinical implications

Embrace Multimodal Analgesia and Regional Techniques

Robust data from systematic reviews and meta-analyses [3,13,17] affirm that regional anesthesia, when feasible, reduces both acute pain and the likelihood of persistent opioid use. Adjuncts like ketamine, dexmedetomidine, IV acetaminophen, and NSAIDs [15] should be first-line components of multimodal protocols. Institutions should invest in training and infrastructure to expand access to nerve blocks, particularly in high-risk surgical settings.

Continue MOUD When Possible

Emerging consensus, including guidelines from Fiellin (2022) and Goel et al. (2019), supports the continuation or strategic adjustment of medications for opioid use disorder during the perioperative period [2,11]. Discontinuing buprenorphine or methadone can destabilize recovery and increase the risk of relapse [17]. Collaboration with addiction specialists is essential when tailoring perioperative plans.

Address Vulnerabilities and Psychosocial Risk Factors

Data from Quaye et al. (2025) and Hah et al. (2017) show that psychiatric comorbidities, smoking, obesity, and other substance use disorders increase the risk of postoperative opioid misuse [6,9]. Pain plans should incorporate psychosocial interventions, anxiety management, and connection to ongoing addiction care [4]. Screening tools and early identification of high-risk patients can guide resource allocation and follow-up intensity.

Ethically Navigate OFA and Patient Preferences

OFA may be appropriate for some patients and procedures, as demonstrated in De Cassai et al. (2022), but clinicians must remain vigilant to the ethical challenges of restricting opioid options. Informed consent, respect for autonomy, and individualized care planning are non-negotiable [14]. Balancing nonmaleficence with patient empowerment is especially critical in stigmatized populations like those with OUD.

Build Institutional Support and Clinical Comfort

Clinician hesitancy and discomfort, as identified in Carvalho et al. (2019), must be addressed through education, clear protocols, and interdisciplinary support. Pain teams, anesthesiologists, addiction specialists, and nurses should collaborate in a multidisciplinary fashion to deliver cohesive, stigma-free care [16].

Call for Research and Policy Alignment

Gaps in the evidence base remain. Conflicting findings, such as those between Chung et al. (2025) and earlier regional anesthesia studies, highlight the need for more nuanced research on which interventions work for whom and in which contexts [18]. Policies should reflect this evolving understanding, ensuring that patients with OUD are not subjected to overly rigid or paternalistic care.

## Conclusions

Patients with OUD deserve pain management that is not only effective but also compassionate and ethical. By integrating multimodal strategies, preserving access to MOUD, identifying vulnerabilities, and centering patient autonomy, we can reduce harm while respecting dignity. The path forward lies not in choosing between analgesia and recovery, but in committing to both, fully and without compromise. Undertreatment of pain, along with stigma-driven care, are forms of harm that contradict the principles of beneficence, nonmaleficence, and justice. Clinicians who embrace these principles will not only improve perioperative outcomes but also contribute to recovery and positive long-term outcomes, reducing relapse risk and strengthening patient trust in healthcare.
